# Size at emergence improves accuracy of age estimates in forensically-useful beetle *Creophilus maxillosus* L. (Staphylinidae)

**DOI:** 10.1038/s41598-018-20796-1

**Published:** 2018-02-05

**Authors:** Szymon Matuszewski, Katarzyna Frątczak-Łagiewska

**Affiliations:** 1 Laboratory of Criminalistics, Adam Mickiewicz University, Św. Marcin 90, 61-809 Poznań, Poland; 2 Department of Animal Taxonomy and Ecology, Adam Mickiewicz University, Umultowska 89, 61-614 Poznań, Poland

## Abstract

Insects colonizing human or animal cadavers may be used to estimate post-mortem interval (PMI) usually by aging larvae or pupae sampled on a crime scene. The accuracy of insect age estimates in a forensic context is reduced by large intraspecific variation in insect development time. Here we test the concept that insect size at emergence may be used to predict insect physiological age and accordingly to improve the accuracy of age estimates in forensic entomology. Using results of laboratory study on development of forensically-useful beetle *Creophilus maxillosus* (Linnaeus, 1758) (Staphylinidae) we demonstrate that its physiological age at emergence [i.e. thermal summation value (*K*) needed for emergence] fall with an increase of beetle size. In the validation study it was found that *K* estimated based on the adult insect size was significantly closer to the true *K* as compared to *K* from the general thermal summation model. Using beetle length at emergence as a predictor variable and male or female specific model regressing *K* against beetle length gave the most accurate predictions of age. These results demonstrate that size of *C. maxillosus* at emergence improves accuracy of age estimates in a forensic context.

## Introduction

There are several methods for post-mortem interval (PMI) estimation based on insect evidence. Most frequently, PMI is approximated based on the age of immature insects sampled from a cadaver. Usually the minimum PMI is being predicted, however the case circumstances (e.g. the probability of myiasis) may change the interpretation^1^. Insect age is estimated using laboratory-derived developmental data and temperature data specific for the decomposition site^2–4^. There are many factors affecting accuracy with which insect age is estimated in a forensic context^5^. Within species variation in development time is one of the largest importance^6,7^. Substantial intraspecific variation of development was revealed in many forensically useful insects^8–14^. Moreover, comparison of the same species studies demonstrated substantial between-study variation in thermal summation constant (*K*) and base temperature (*T*_*b*_)^15–18^.

Several sources of the intraspecific variation in development time were identified in forensically useful insects. Gallagher *et al*.^19^ demonstrated for a blowfly *Lucilia sericata* (Meigen, 1826) that some part of this variation results from differences between local populations. Similar findings were reported for other blowflies, *Chrysomya megacephala* (Fabricius, 1794)^20^ and *Cochliomyia macellaria* (Fabricius, 1775)^21^. Moreover, differences in development time between females and males were reported for *L. sericata*^22^, a phorid fly *Megaselia scalaris* (Loew, 1866)^23^ and a staphylinid beetle *C. maxillosus*^24^. Another source is the precocious egg development, resulting in some eggs from the batch hatching earlier, which occur commonly in sarcophagid flies and less frequently in calliphorid flies^5,25^. Several exogenous determinants of development time were identified as well; for example, quality and quantity of food^6,26–30^ or intra and interspecific competition^31–33^.

Although intraspecific variation in development time of forensically useful insects is usually large, there were just a few suggestions how these variation may be taken into account while estimating insect age (but see^34^). Regarding geographical variation several authors proposed to use, wherever possible, local developmental data^16,35^. It is, however, unclear what are the error rates resulting from geographical mismatch between laboratory and crime scene insects. Richards *et al*.^17^ demonstrated that *K* for a blowfly *Chrysomya albiceps* (Wiedemann, 1819) was proportional to geographic latitude. Based on this finding they have suggested that it might be possible to develop a model useful for *K* and *T*_*b*_ estimation at any latitude^17^. Unfortunately, no such model has been derived for any forensically useful species. Another suggestion resulted from studies of sex-specific developmental patterns in forensically useful insects^22,24^. Picard *et al*.^22^ suggested that sex-specific developmental data could be used to increase the accuracy of insect age estimates and reduce error rates in minimum PMI estimates. However, recent study with forensically-useful beetle *C. maxillosus* did not support the use of sex-specific developmental models in forensic entomology, as despite significant differences in development time between males and females of *C. maxillosus*, there was no gain in the accuracy of age estimates using sex-specific developmental models^24^. There is also a general recommendation that while estimating insect age, one should use developmental models derived in similar conditions compared to the case conditions^4,16^. Wells & LaMotte^36^, however, indicated that not all differences between laboratory and crime scene conditions are of practical importance. They showed that despite highly significant effect of food type on larval growth rate, the developmental data for *C. megacephala* larvae grown on liver gave quite accurate age predictions for larvae grown on heart tissue^36^. Unfortunately, performance of the predictive model is usually unknown, the importance of the factor may still be evaluated based on its effect size on the relevant developmental parameter. Summarizing, none of the above suggestions is supported by the empirically demonstrated gain in the accuracy of insect age estimates.

Here we test the concept that insect size at emergence is a good predictor for development time and accordingly it may be used to improve the accuracy of insect age estimates in forensic entomology. A correlation between size at maturity and development time is widespread in insects^37,38^. Among herbivorous or predatory insects negative correlation was reported with high regularity, whereas the positive correlation was limited to parasitoids^38^. It is therefore reasonable to assume that insect size at emergence may be used to estimate its age and here this assumption is tested with forensically useful beetle *C. maxillosus*. We predict that 1) thermal summation value (*K*) needed by individual beetles of *C. maxillosus* to reach the adult stage is related to the size of beetles at emergence, 2) using the insect size to estimate *K* may significantly improve the accuracy of insect age estimates in forensic entomology, and 3) the relationship between insect size and *K* needed for emergence will be represented with higher accuracy by separate models for males and females than the pooled model. To test these predictions we used results of developmental experiment, in which immature *C. maxillosus* were reared using standardized laboratory protocol, at different constant temperatures and optimal food conditions, with monitoring of development time and determination of insect size and sex at emergence.

*C. maxillosus* is a predatory beetle regularly visiting and breeding in large vertebrate cadavers including humans^39–43^. Forensically useful developmental models were recently derived for this species^10,24^. Robust methods for classifying larval instars were developed as well^44^. It was also demonstrated that an interval preceding appearance of adult or larval stages of *C. maxillosus* on cadavers (i.e. the pre-appearance interval, PAI) is strongly related to temperature^45^ and may be accurately estimated using temperature methods for PAI^46,47^. Accordingly, *C. maxillosus* may be regarded as useful for PMI estimation using entomological methods. As for the topic of this article, previous studies revealed substantial variation in size of adult *C. maxillosus*^48^ and in the duration of third larval and pupal developmental stages^10,24^.

## Results

### Relationship between physiological age and size at emergence in *C. maxillosus*

**Figure 1:**
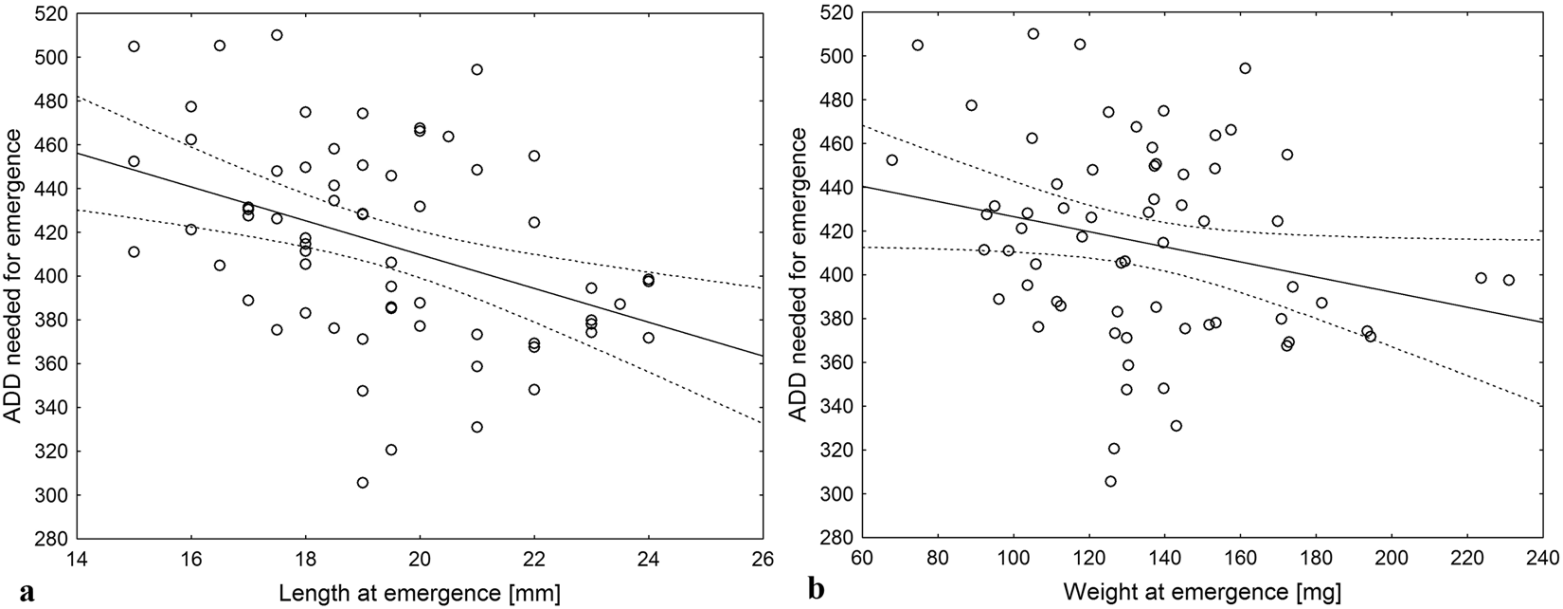
The relationship between adult *C. maxillosus* length (**a**) or weight (**b**) at emergence and thermal summation values needed for emergence (ADD over 11.58 °C). Solid line – linear regression model, dotted lines – confidence limits.

**Figure 2:**
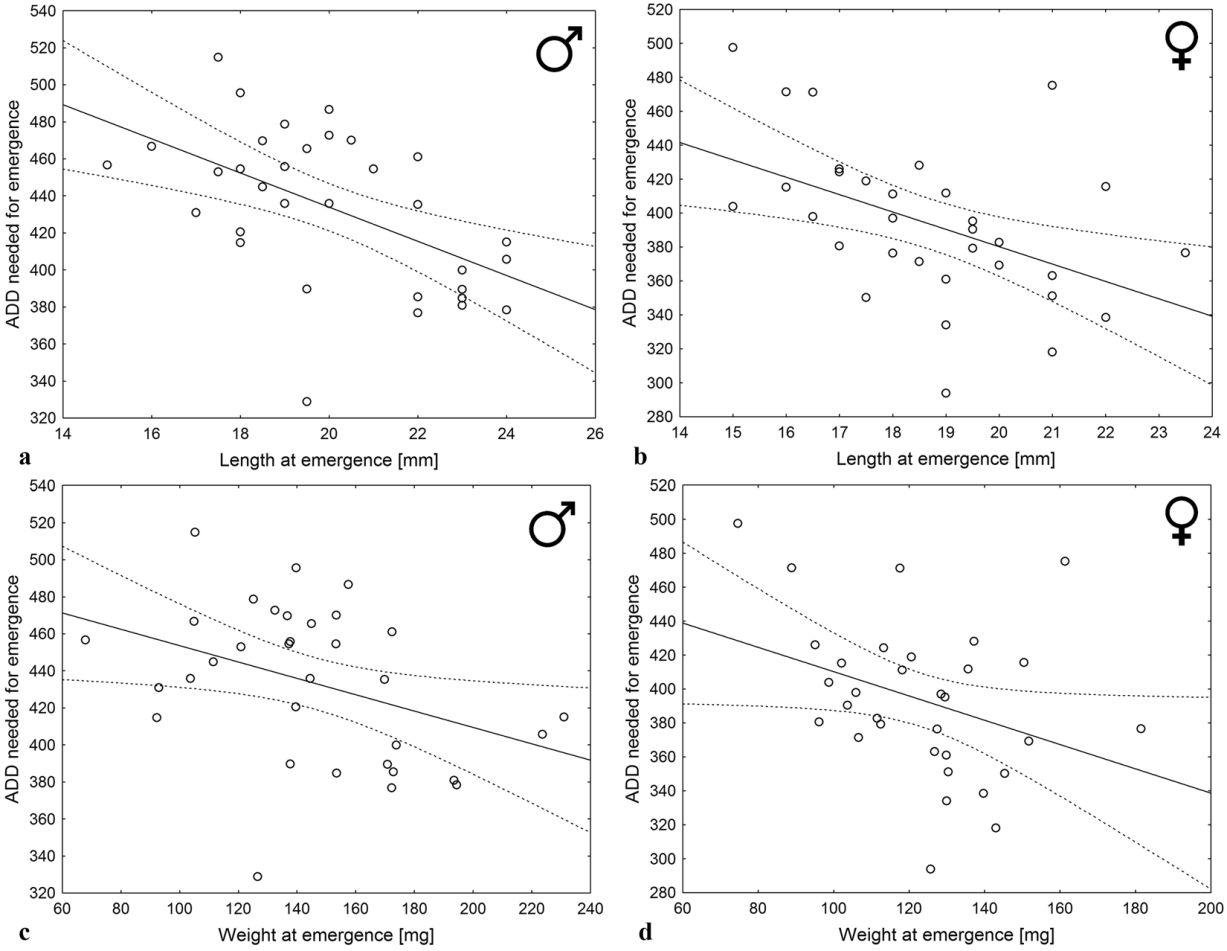
The relationship between adult *C. maxillosus* length (**a**,**b**) or weight (**c**,**d**) at emergence in male (**a**,**c**) or female (**b**,**d**) sample and thermal summation values needed for emergence (ADD over 11.43 °C for males and over 11.81 °C for females). Solid line – linear regression model, dotted lines – confidence limits.

Thermal summation values (*K*) needed for emergence of *C. maxillosus* fell with an increase of beetle size at emergence (Figs 1, 2, Table 1). Depending on the model, beetle size explained from 6% to 28% of variation in *K* needed to reach the adult stage (Table 1). The relationship between *K* and insect size was represented with larger accuracy by models calculated separately for males (Fig. 2a,c) and females (Fig. 2b,d) than models calculated for the pooled sample (Fig. 1). Beetle length at emergence explained more variation in *K* than beetle weight at emergence (Table 1).

**Table 1:** Linear regression models for the relationship between length or weight of adult *C. maxillosus* at emergence and thermal summation values (accumulated degree days, ADD) needed to reach the adult stage

Model	Response variable (*y*)	Predictor variable (*x*)	Model	*N*	*F*	*P*	*r* ^2^
Pooled	ADD over 11.58	Length	*y* = 564.31–7.722*x	65	12.08	0.0009	0.16
	ADD over 11.58	Weight	*y* = 461.17–0.3456*x	65	4.03	0.049	0.06
Male-specific	ADD over 11.43	Length	*y* = 618.33–9.223*x	33	12.34	0.0014	0.28
	ADD over 11.43	Weight	*y* = 497.84–0.4423*x	33	5.37	0.027	0.15
Female-specific	ADD over 11.81	Length	*y* = 584.76–10.23*x	32	8.41	0.0069	0.22
	ADD over 11.81	Weight	*y* = 481.85–0.7165*x	32	4.21	0.049	0.12

### Estimation of physiological age based on insect size at emergence in *C. maxillosus*

**Figure 3:**
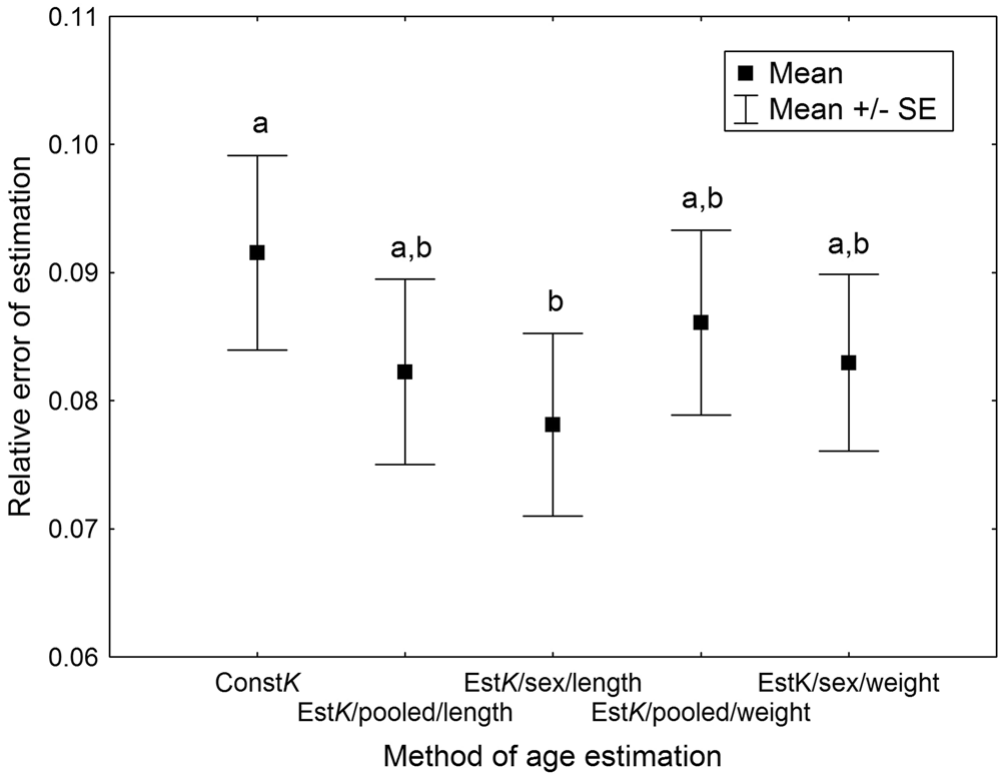
Relative error of *C. maxillosus* physiological age (*K*) at emergence estimation using different methods. Const*K* – the use of constant *K* (from the general thermal summation model, 417.33 ADD over 11.58 °C after Frątczak-Łagiewska & Matuszewski^24^); Est*K/*pooled/length – estimation of *K* using the model for the pooled sample and beetle length at emergence as the predictor variable; Est*K/*sex/length – estimation of *K* using models for females and males and beetle length at emergence as the predictor variable; Est*K/*pooled/weight – estimation of *K* using the model for the pooled sample and beetle weight at emergence as the predictor variable; Est*K/*sex/weight – estimation of *K* using models for females and males and beetle weight at emergence as the predictor variable. Different letters denote significant differences in pairwise comparisons (absolute differences between mean ranks were significant at 5% level of significance, if they were larger than 0.477).

**Figure 4:**
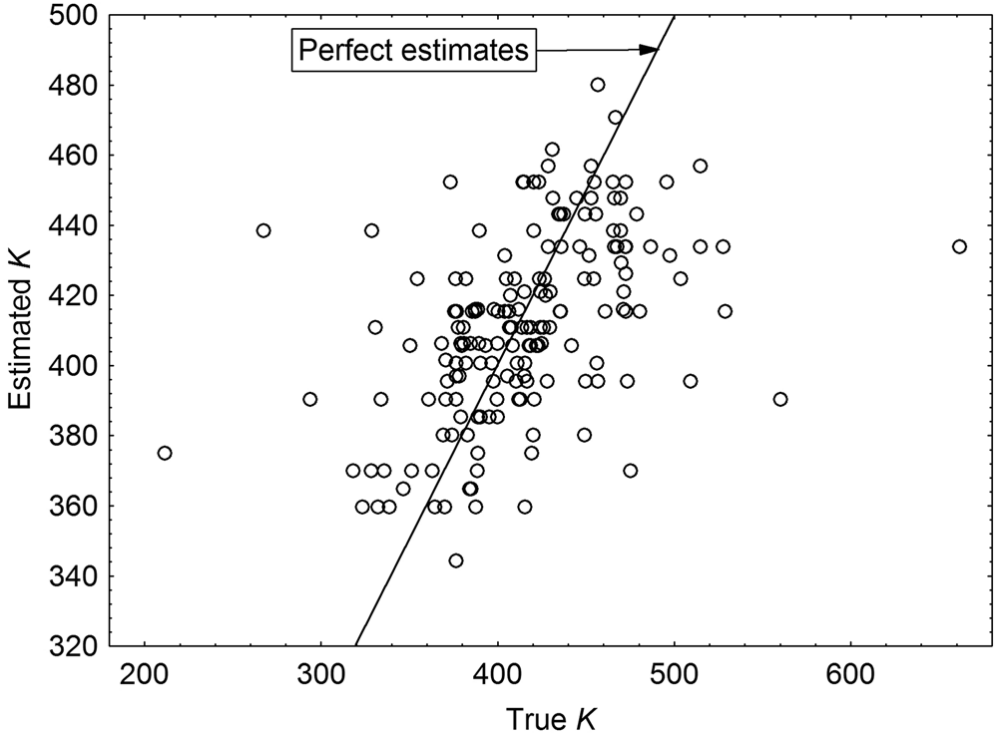
Estimates of physiological age (*K*) at emergence in *C. maxillosus* plotted against the true age. *K* was estimated using sex-specific models regressing *K* and beetle length at emergence. Solid line – hypothetical line representing perfect estimates.

**Figure 5:**
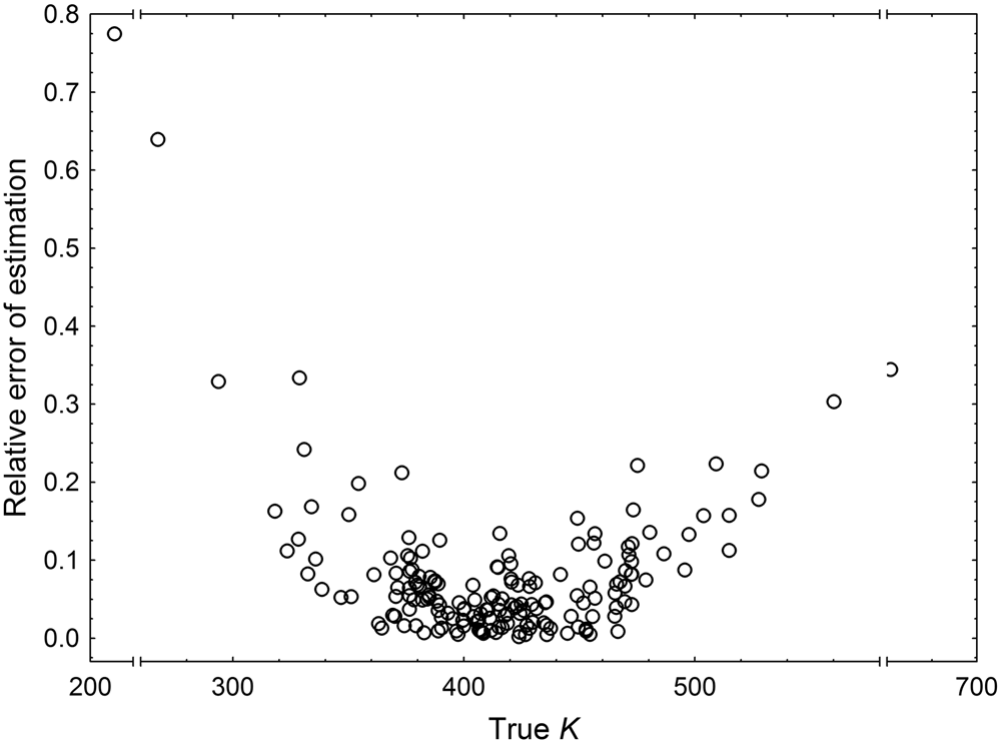
Relative error in estimates of physiological age (*K*) at emergence of *C. maxillosus* plotted against the true age. *K* was estimated using sex-specific models regressing *K* and beetle length at emergence.

There were significant differences in the relative error of estimation between methods used to estimate physiological age of *C. maxillosus* at emergence (Friedmann ANOVA, *χ*^2^ = 17.33, *P* = 0.0017, N = 108, 51 males and 57 females; Fig. 3). *K* from the general thermal summation model (i.e. 417.33 accumulated degree days [ADD] over 11.58 °C after [Frątczak-Łagiewska & Matuszewski^24^]) represented true *K* with the average difference of 9% (Fig. 3). Estimation of *K* based on *C. maxillosus* size, gave more accurate representation for the true *K* irrespective of the method used for the estimation (Fig. 3). Using beetle length at emergence as a predictor variable and sex-specific models resulted in the best representation of the true *K* (Fig. 3). While estimating *K* with this method, for the true *K* below 380 ADD, estimates were mostly overestimations, whereas for the true *K* above 440 ADD, estimates were mostly underestimations (Fig. 4). If the true *K* was below 380 ADD, error of estimation increased with the decrease of *K*, and for the true *K* above 440 ADD error increased with the increase of *K* (Fig. 5).

## Discussion

As expected, physiological age at emergence of *C. maxillosus* was related to its size. The negative relationship is in line with the pattern revealed in the meta-analysis of Teder *et al*.^38^, who demonstrated that it is prevalent in predatory insects and *C. maxillosus* predatory feeding habits has been very well documented^49,50^. While validating our concept, no matter what method was used for the estimation, *K* predicted based on the adult insect size was significantly closer to the true *K* as compared to the *K* from the general thermal summation model. This finding demonstrates that size of *C. maxillosus* at emergence improves accuracy of physiological age estimates for this species. Because many other forensically-useful insects reveal large within-species variation of insect size, e.g. puparia of *L. sericata*^51^, adult stages of larder beetle *Dermestes maculatus* (DeGeer, 1774)^52^ or a silphid beetle *Necrodes littoralis* (Linnaeus, 1758)^48^, the method described here may have much wider applicability. Our results indicate that length of adult *C. maxillosus* is more useful for prediction of age than weight. This finding reflects larger intra and inter individual variation in weight of adult insects.

Usage of the models representing association between *K* and insect size involves several basic concepts related to age and size. One of the most important is the temperature-size rule, the taxonomically widespread pattern of larger body size at lower developmental temperatures which holds for natural populations and laboratory reared ectotherms^53,54^. As a rule ectotherms (e.g. insects) in colder conditions grow slower but are larger at maturity, whereas at higher temperatures they grow faster but are smaller^55,56^. The former conditions should result in proportionally more time needed for the emergence, whereas the latter conditions should be accompanied with proportionally less time. At lower temperatures one may expect that larger *K* will be associated with larger body size of an insect, and at higher temperatures smaller *K* with smaller body size. Current results contradict such a simple interpretation, as the relationship between *K* and size of *C. maxillosus*, analyzed in the whole temperature range, was clearly negative. Our results are however in line with predictions of optimality models^38,57–59^, in case of which optimal conditions are associated with larger size and shorter development and non-optimal conditions with smaller size and longer development. It is thus possible that in the range of optimal temperatures the relationship between *K* and insect size is distinctly negative, whereas inclusion of non-optimal (lower and higher) temperatures may weaken the negative slope of the relationship. This interpretation suggests that the usage of separate models for optimal and non-optimal temperatures might further improve the accuracy of insect age estimates in forensic or other scenarios. To test this possibility further studies are necessary.

Optimality models indicate that poor resources cause insects to emerge at smaller sizes but after longer development and vice-versa at optimal resources. Such tradeoffs between age and size are common in insects^38^ and were also reported for several forensically-useful species, with depletion of cadaver tissues or insect overcrowding on cadavers resulting regularly in stunted larvae^31,32^. Although we used uniform diet across the temperatures and therefore did not catched the diet-related variation in *K* and size, we feel that the models derived in the current study are reasonably universal and therefore may be used for prediction irrespective of the specimen feeding history. To justify this assertion further studies are however needed. Similar limitations may be posed by differences in development between local populations which are widespread in insects, forensically useful as well^19–21^, and usually have some genetic component. In forensic entomology several approaches may tackle these complications. The most valid but at the same time the most laborious would be to diversify empirical foundations for the models, for instance by using insects from different populations, reared at different temperatures and various diets. Another solution is to test current and future models in prediction tasks with insects from different populations (with different geographic origins or different diets). While recently there has been substantial progress in forensic entomology, particularly in understanding foundations of the discipline^60^, many of the findings have not translated into forensically useful techniques and we feel that there are still large areas of the field where substantial progress is needed and possible.

Some authors suggested that insect age estimates may be more accurate through the use of sex-specific developmental data^22^. Recent study revealed however no gain in the accuracy of age estimates while using separate thermal summation models for females and males^24^. Current results demonstrate that insect sex may be useful, yet it needs to be analyzed in conjunction with size. Different relationship between *K* and size in the case of males and females as well as the larger increase in the accuracy of *K* estimates while using sex-specific models indicate that sex may be regarded as useful co-predictor of insect age, at least in the case of *C. maxillosus* and in a forensic context.

Although there is a tendency in applied entomology to consider *K* as the species-specific constant, a large body of data contradicts this view. Current results support the notion of *K* as a characteristic of high intraspecific variability. Accordingly, we postulate that *K* should be predicted for an insect evidence and forensic entomologists should develop models useful for this purpose. Sex-specific models regressing *K* and insect length at emergence significantly improved the accuracy of age estimates in the case of *C. maxillosus*, however there is still the need to search for other traits useful for *K* prediction.

**Figure 6:**
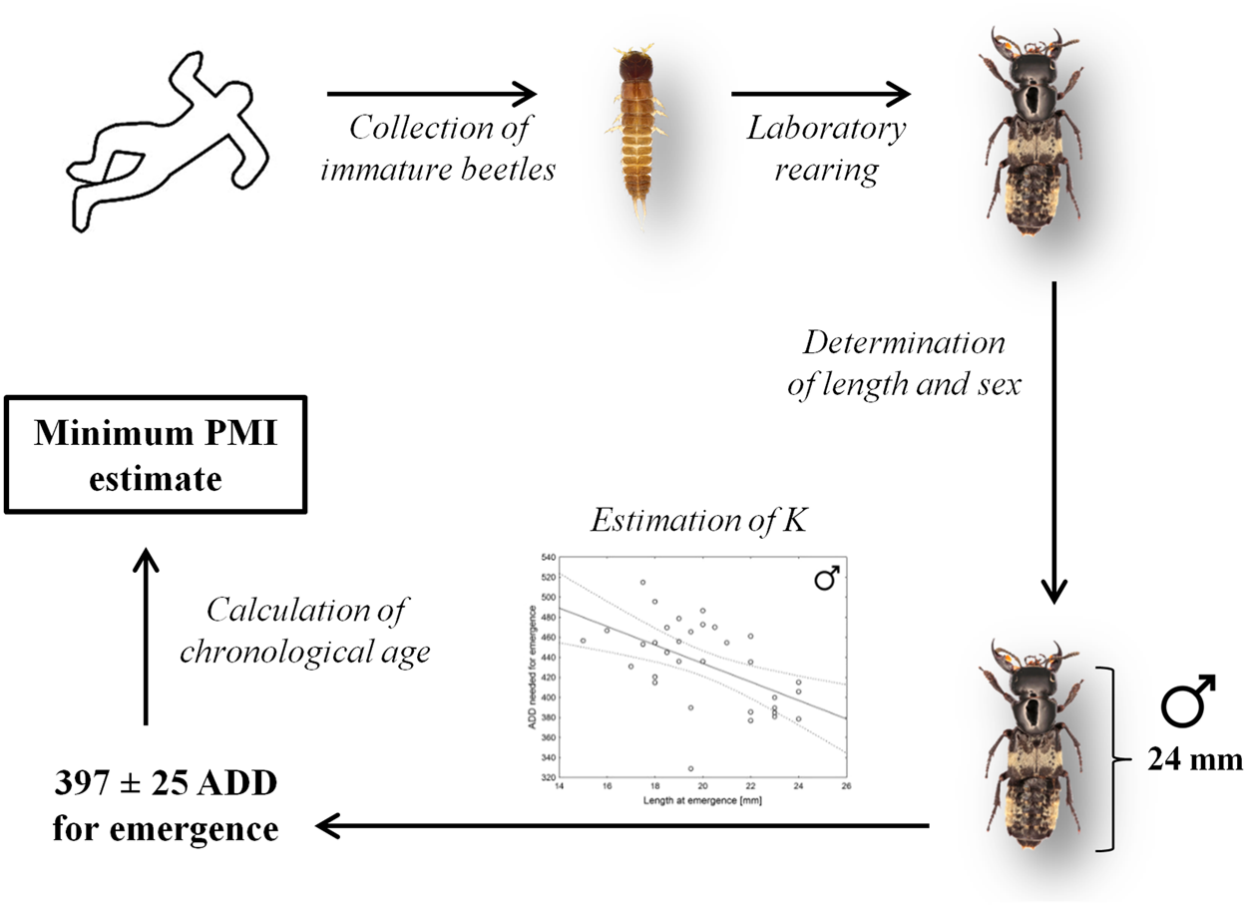
A schematic representation for the implementation of the method in forensic entomology. Anna Mądra-Bielewicz is the copyright holder of the *C. maxillosus* pictures used in this figure.

In order to implement the method, live immature beetles should be sampled at a crime scene and reared to the adult stage in the laboratory (Fig. 6). At emergence insect length should be measured and sex determined. They may be used to estimate *K* for the crime scene beetles using models from the current article. Then, estimated *K* should be used to approximate insect chronological age at the moment of sampling (and eventually minimum PMI) using protocols developed in forensic entomology. Because the method needs live specimens, it may be combined with the approach for aging insects developed by Marchenko^61^.

### Self-critique

#### Strength of the relationship between physiological age and size of C. maxillosus

*R*^2^ derived for the best models indicate that for female *C. maxillosus* 22% of variation in *K* may be explained by variation in adult insect size, whereas in the case of male beetles it is 28%. Although for both sexes about a quarter of variation in *K* was due to the adult insect size, the models may and should be refined by future studies. Despite their weaknesses, in the validation part of the study the models outperformed the current routine of using constant *K*. This is the most important finding of the study. Current results demonstrate that simple models for physiological age and insect physical traits (here size) may significantly improve accuracy of insect age estimates in forensic or more general applied entomology.

#### The gain in the accuracy of K representation resulting from its estimation based on the adult insect size

Although estimation of *K* using our best model gave significantly more accurate representation for the true *K* as compared to the constant *K* from the thermal summation model, the gain in the accuracy was rather minor. The thermal summation constant represented the true K with the average error of 9.2% (Fig. 3), e.g. for the *K* of 417 ADD it gives an error of 38.4 ADD over 11,58 °C (e.g. about 6 days at average temperature of 18 °C). *K* estimated using adult insect length as the predictor variable and sex-specific models represented the true *K* with the average error of 7.8% (Fig. 3), e.g. for the *K* of 417 ADD it gives an error of about 32.5 ADD over 11,43 °C for a male beetle (e.g. about 5 days at average temperature of 18 °C). The gain of about 6 ADD (i.e. about a day at temperature of 18 °C) is not much (at 18 °C 417 ADD will accumulate over 11.58 °C after about 65 days). We think that this minor improvement was a result of large similarity between training and validation samples of insects used in the current study. Both samples originated from the same laboratory experiment and not surprisingly the baseline accuracy (the one associated with *K* from the general thermal summation model) was high leaving little area for improvement. We feel that validating our models with a more diverse insect sample (e.g. beetles with the diverse nutritional history) would reveal a larger gain in the accuracy of *K* estimates. Current method could be refined also by including other physical traits alongside insect size at maturity. This article therefore should be treated as the first step to build a multi-factor model for the estimation of *K*.

## Materials and Methods

### Rearing procedures

A colony of adult *C. maxillosus* was maintained in the laboratory at room temperature and humidity (20–22 °C, 50–60%). Insects were kept in plastic containers (30,4 × 20 × 20,1 cm) on a damp soil and were fed with puparia or third instar larvae of blowflies. Colonies were established in spring of 2015 and 2016 using beetles sampled from rabbit carcasses exposed in the Biedrusko military range (Western Poland, Europe; 52 31’N, 16 55’E). Throughout the study the colony consisted of 25–30 beetles, new beetles sampled in the field or reared in the laboratory were used to reinstate the colony. Between 10 and 20 new beetles were added to the colony per month. To minimize effect of laboratory inbreeding, we used eggs from field-captured insects or the first laboratory generation.

Immature beetles were reared individually (80 ml containers with 1,5 cm of soil for the 1^st^ and the 2^nd^ instar larvae, 120 ml containers with 5 cm of soil for the 3^rd^ instar larvae and pupae) in temperature chambers (ST 1/1 BASIC or +, POL-EKO, Poland) at constant temperature and humidity (15, 17.5, 20, 22.5, 25, 27.5 and 30 °C; air humidity: 60–70%; photoperiod (h): 12:12 (L:D)). Individual rearing conditions are close to the natural conditions for this species and make it possible to monitor individual insects throughout their development. Due to the difficulties in sampling eggs of *C. maxillosus*^24^ and their high mortality in the laboratory conditions^10^, beetles were kept in separate containers from the onset of larval stage. In order to obtain eggs the entire adult colony was transferred into a three liter container with soil and kept at 20–22 °C for four hours. Afterwards, containers (with no adult insects inside) were placed in incubators with the given temperature and were inspected for the presence of first instar larvae every 10% of the average egg stage duration (inspections started after 70% of the average egg stage duration). Forty freshly hatched larvae were used per temperature. Two or three temperatures were studied simultaneously with random assignment of insects to temperatures. Larvae were fed once a day with third instar larvae of blowflies.

Transitions between developmental stages (i.e. hatching, first ecdysis, second ecdysis, pupation and adult emergence) were monitored in all insects at intervals equal to 10% of the average stage duration. Half of the beetles were measured and weighed throughout larval and pupal development. At emergence beetle sex was identified based on the shape of the eighth abdominal sternite. Adult beetle length (from the anterior margin of the clypeus to the posterior margin of the last abdominal segment) was measured *in vivo* using geometrical micrometer^62^ after beetle became fully erect in a 1.5 ml eppendorf tube. Weight of adult beetles was measured *in vivo* in an eppendorf tube using analytical balance (AS 82/220.R2, Radwag, Poland).

### Data analyses

Base temperature (the temperature below which development stops^16^) for the total immature development in the case of the male-specific model is 11.43 °C, in the case of the female-specific model it is 11.81 °C and in the case of the pooled sample model it is 11.58 °C^24^. Thermal summation values (*K*) needed to reach the adult stage were calculated over these temperatures for individual beetles and then were regressed against adult beetle length or weight at emergence using linear regression. Insect length or weight at maturity were used as predictor variables and *K* as a response variable. Growth of insects slows (and eventually stops) after a larva surpasses a critical weight and several molecular mechanisms are responsible for insect size assessment and control at critical weight^63,64^. Therefore, the cessation of growth (and eventually *K*) depends on the insect size and not vice-versa. Regression analyses were used to test whether *K* and size are related to each other and what is the effect size of insect length or weight on *K*. Because we predicted that the relationship between *K* and size will be closer when analyzed separately for males and females as compared to the pooled sample, regression analyses were performed for the female sample, the male sample and the pooled sample. Models for females and males were based on the sample consisting of five randomly chosen males or females per temperature (in total 33 males and 32 females; at 15 °C there were just 2 females, at 30 °C there were just 3 males). Models for the pooled sample were based on 65 insects. Rest of the specimens (i.e. 51 males and 57 females) were used to test the concept (due to high mortality, 15 and 30 °C were underrepresented in the validation sample). The current regression models were used to predict *K* needed for emergence based on the length or weight at emergence of insects used in the validation. Then, estimated *K* were compared to the true *K*, and resultant error rates were analyzed across methods using the Friedman ANOVA. Analyses were made using Statistica 12 (Dell, Inc., 2013) at 5% level of significance.

### Data availability

The datasets generated and/or analyzed during the study are available from the corresponding author on a reasonable request.
